# Pharmacokinetics of CAR T cells in multiple myeloma – the good, the bad, and the ugly

**DOI:** 10.1038/s41375-026-02933-2

**Published:** 2026-03-31

**Authors:** Maximilian Merz, Eric Jurgens, David Fandrei, Saad Z. Usmani, Sham Mailankody

**Affiliations:** 1 Myeloma Service and Cellular Therapy Service, Memorial Sloan Kettering Cancer Cetner, New York, NY USA; 2 Department of Hematology, Cellular Therapy, Hemostaseology and Infectiology, University Hospital of Leipzig, Leipzig, Germany

**Keywords:** Translational research, Cancer immunotherapy, Myeloma

## Introduction

More than a decade after the initial clinical reports of chimeric antigen receptor (CAR) T-cell therapy emerging from early-phase trials and individual case studies [1–3]. CAR T cells directed against B-cell maturation antigen (BCMA) have now evolved into an established standard of care for patients with relapsed or refractory multiple myeloma (RRMM) [4].

Idecabtagene vicleucel (ide-cel) received regulatory approval based on the KarMMa-1 and KarMMa-3 studies for patients who have undergone at least two prior lines of therapy and have been exposed to a CD38-targeting antibody, a proteasome inhibitor (PI), and an immunomodulatory agent (IMiD) [5–8]. In parallel, informed by the CARTITUDE-1 and CARTITUDE-4 trials, ciltacabtagene autoleucel (cilta-cel) is approved for use after a single prior line of therapy in patients previously exposed to a PI and an IMiD and whose disease is refractory to lenalidomide [9–12].

Although highly efficacious and associated with treatment-free intervals and improved quality of life compared with conventional doublet and triplet regimens in multiple myeloma, ide-cel has been consistently outperformed by cilta-cel in recent real-world analyses from the United States and the European Union [13–17].

These real-world analyses demonstrated higher overall and complete response rates, as well as longer progression-free and overall survival, with cilta-cel compared to ide-cel. However, they also indicated signals of increased rates of cytokine release syndrome (CRS) and immune effector cell-associated neurotoxicity syndrome (ICANS) associated with cilta-cel.

Importantly, a recent update from CARTITUDE-1 showed that more than 30% of heavily pretreated patients remained progression-free five years after receiving cilta-cel [11]. While their prior therapies had typically controlled disease for only a few months, this long-term disease control highlights the potential for cilta-cel to provide durable and possibly curative benefit.

**Fig. 1:**
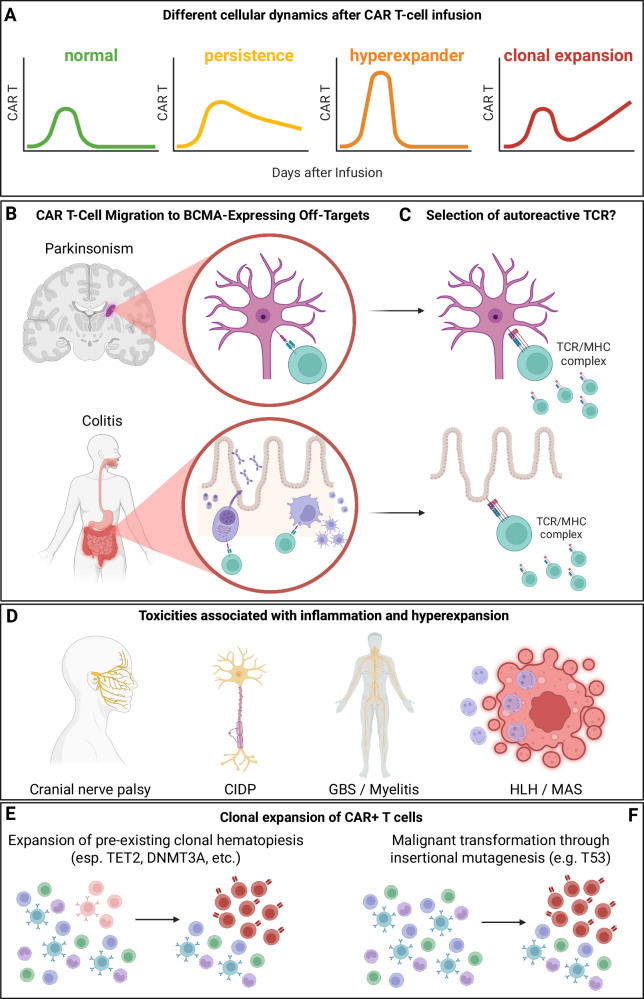
Overview of CAR T-cell pharmacokinetics, off-target toxicities, and clonal risks in multiple myeloma. **A** *Representative CAR T-cell kinetic profiles after infusion*. Four distinct patterns of cellular dynamics are shown: normal expansion with contraction that usually occurs around day 60 after infusion (green curve); persistent expansion with extended low-level CAR T-cell presence beyond day 60 (orange curve); hyperexpansion, characterized by rapid, high-magnitude proliferation usually with lymphocyte counts >3/nl associated with delayed toxicities (yellow curve); and clonal expansion, reflecting pathological, antigen-independent proliferation that may signal secondary CAR-positive T-cell lymphoma (red curve). **B** *Off-target migration of CAR T cells to BCMA-expressing healthy tissues*. BCMA expression on non-malignant cells can facilitate CAR T-cell infiltration into sensitive anatomical sites. Examples shown include dopaminergic neurons in the basal ganglia (associated with parkinsonism) and immune and epithelial cells in the gastrointestinal tract (associated with IEC-colitis). These off-target interactions may initiate tissue inflammation or trigger downstream immune dysregulation. **C** *Potential selection or activation of autoreactive T-cell receptors (TCRs)*. In some patients, CAR T-cell expansion may be accompanied by recruitment or proliferation of autoreactive T cells. This process may contribute to delayed toxicities and might explain why these side effects do not occur more often and with all BCMA-targeted therapies. **D** *Toxicities linked to hyperinflammation and hyperexpansion*. Clinical manifestations associated with excessive CAR T-cell expansion include cranial nerve palsies (CNP), CIDP, GBS or myelitis, and HLH/MAS. These syndromes often occur days to weeks after peak expansion, are frequently preceded by CRS or ICANS, and may require aggressive monitoring and intervention. **E** *Clonal expansion driven by pre-existing hematopoietic mutations*. Expanding CAR T-cell populations may preferentially amplify clones harboring mutations linked to clonal hematopoiesis, notably *TET2*, *DNMT3A*, or related genes. Such selection can give rise to aberrant CAR-positive T-cell populations detectable in blood or tissue, reflecting one mechanism of secondary CAR-positive T-cell lymphoma described in recent reports. **F** *Malignant transformation through vector-related events*. In rare cases, insertional mutagenesis, such as monoallelic integration of a CAR transgene into *TP53* may drive uncontrolled CAR T-cell proliferation. This represents another route to secondary lymphoma and underscores the need for genomic and phenotypic evaluation when CAR-positive clones persist or expand unexpectedly. (created with biorender).

Since the approvals of cilta-cel and ide-cel, tens of thousands of patients have been successfully treated outside of clinical trials. Although more common short-term toxicities, such as CRS, ICANS, infections, and cytopenias are typically manageable and rarely compromise the long-term therapeutic benefit of CAR T-cell therapy in myeloma, there is increasing recognition of rare but severe adverse events. These include immune-effector-cell–associated enterocolitis [18, 19]. delayed neurotoxicities, such as movement and neurocognitive treatment-emergent events (MNTs) [20], including parkinsonism [21]. Guillain–Barré syndrome (GBS) [22]. chronic inflammatory demyelinating polyneuropathy (CIDP) [23]. and myelitis; as well as hemophagocytic lymphohistiocytosis (HLH) [24]. and secondary CAR-positive T-cell lymphomas [25–29] (Fig. 1).

The pathogenesis underlying these adverse events remains incompletely understood, and both diagnosis and management continue to be challenging. Emerging evidence suggests substantial overlap among several of these conditions [30]. For example, some secondary CAR-positive T-cell lymphomas can present with gastrointestinal (GI) manifestations, and neuroinflammatory phenomena, such as cranial nerve palsies (CNP) may precede or coincide with GBS, including Miller–Fisher variants. In addition, high-grade CRS frequently precedes the development of HLH [31].

Although these conditions represent distinct clinical entities, a recurring theme may offer a promising target for early diagnostic strategies and preemptive intervention: detailed characterization of CAR T-cell pharmacokinetics after infusion. Hyperexpansion [32] and persistent off-target tissue infiltration, as well as (oligo)clonal CAR T-cell proliferation, have been linked to delayed neurotoxicity [33], colitis [34], and secondary malignancies. At the same time, robust and sustained CAR T-cell expansion has also been correlated with improved survival outcomes in myeloma [35, 36]. underscoring the complex balance between therapeutic efficacy and toxicity.

In this perspective, we summarize the current evidence linking CAR T-cell kinetics with clinical outcomes. We highlight potential opportunities for improved diagnostics, therapeutic strategies, and prophylactic interventions. Ultimately, this article provides an integrated view of the ‘good, the bad, and the ugly’ of CAR T-cell expansion in relapsed multiple myeloma.

## The good – robust and persistent expansion is associated with outcomes

CAR T-cell expansion is a critical determinant of efficacy, as it shifts the effector-to-target ratio in favor of the therapeutic cells. Long-term CAR T-cell persistence—extending over a decade—has been documented in patients who achieved durable remission after CAR T-cell therapy [37, 38]. However, recent clinical trials have shown that the mere presence of CAR T cells does not necessarily prevent disease recurrence. It remains unclear whether resistance is driven primarily by myeloma cells acquiring escape mechanisms or by functional impairment of the CAR T cells themselves.

Because CAR T-cell therapy exerts only transient selective pressure, antigen loss, such as BCMA downregulation or deletion—occurs relatively infrequently after treatment, with rates of approximately 5% [39]. This contrasts with the much higher rates reported with continuous bispecific antibody therapy, where BCMA loss can reach up to 50% [39]. Nonetheless, clinical trials of ide-cel and cilta-cel consistently demonstrate that robust and sustained CAR T-cell expansion is associated with superior survival outcomes [5, 40].

### Karmma-1

Pharmacokinetic analyses from KarMMa-1 demonstrated that ide-cel expansion peaked at a median of 11 days post-infusion, with substantial interpatient variability [5]. Higher transgene exposure (area under the curve, AUC within the first 28 days after infusion) occurred more frequently at the 450×10⁶ CAR T cell dose level and was consistently associated with improved clinical outcomes. Patients who responded to therapy exhibited greater CAR T-cell exposure than non-responders, and higher expansion correlated with deeper responses, longer progression-free survival, and more pronounced reductions in soluble BCMA. CAR T cells remained detectable in 59% (*n* = 29) of evaluable patients (*n* = 49) at 6 months and in 36% (four of 11 patients) at 12 months, indicating persistence beyond the acute expansion phase [5].

The development of antidrug antibodies (ADAs), a potential mechanism of CAR T-cell clearance, were analyzed within KarMMa-1. ADAs were not detected in the first 3 months after infusion. ADA prevalence increased from 21% (21 of 102 patients) at month 3 to 65% (34 of 52 patients) at month 12. However, ADA positivity did not affect key pharmacokinetic parameters, including AUC or maximum expansion and ADA had no effect on outcomes [5].

### Karmma-3

In KarMMa-3, pharmacokinetic data were available for 224 of 254 patients. Following infusion, ide-cel reached peak expansion levels at a median of 11 days [7, 41]. Exploratory analyses demonstrated a clear association between higher quartiles of CAR T-cell expansion and longer PFS. Comparable to KarMMa-1, substantial interpatient variability in expansion kinetics was observed, with the lowest quartile of expansion represented across all administered dose levels—suggesting no clear association between the number of infused CAR T cells and subsequent in vivo expansion.

#### CARTITUDE-1

In the initial pharmacokinetic analysis of the CARTITUDE-1 study, the manufactured cell product included both CAR-transduced and non-transduced T cells [40]. Overall transduction efficiency was modest, with a median of 16% (range, 5–32%) CAR-transduced T cells. CAR-expressing CD4⁺ and CD8⁺ T cells were present in similar proportions, with median frequencies of 12% (range, 2–28%) and 6% (range, 2–20%), respectively. The broader T-cell phenotype distribution varied among patients but consistently showed a mixture of central memory and effector memory subsets [40]. Cilta-cel peak expansion was observed between days 12-14 after infusion reaching a median concentration of 730 cells/µL (range 3-13805 cells/µL). Detectable levels of Cilta-cel in the peripheral blood were observed for a median of 100 days (range 20–912 days). Most likely due to the high reported efficacy and duration of response after Cilta-cel treatment, there was no direct connection between expansion or persistence and response. Peak expansion was characterized by a marked skewing toward CAR⁺ CD8⁺ T cells (CD4:CD8 ratio of 0.29). At this time point, the majority of both CD4⁺ and CD8⁺ CAR T cells displayed a central memory phenotype (median proportions of >90%, respectively). Progression-free patients for ≥5 years versus those with PD within 5 years had higher effector to target ratio and CAR + T-cell peak expansion (961 cells/μL versus 450 cells/μL) [11]. Patients who remained progression-free tended to exhibit more CD4 central memory CAR T cells and showed enrichment for activation markers including CD38, CD25, and PD-1.

#### CARTITUDE-4

Recently, pharmacokinetic correlative analyses from 176 patients treated with cilta-cel in CARTITUDE-4 were reported [42]. Overall, CAR T-cell expansion and persistence in CARTITUDE-4 were consistent with CARTITUDE-1. Cilta-cel demonstrated rapid expansion, with mean peak CAR T-cell expansion of 1451 cells/µL (SD 6169), occurring at a median of 12.9 days (range 7.8–222.8). Overall exposure during the first 28 days was significant, with a mean AUC of 11,710 day×cells/µL (SD 56,994). CAR T cells remained detectable for a median of 57 days, with a range of 13 to 631 days [42]. Peak expansion and AUC were not different between patients in (stringent) complete response compared to all other responses. sBCMA levels declined in all patients, becoming undetectable at a median of 56 days. At disease progression, sBCMA levels rose again, while no corresponding re-expansion of CAR T-cells was observed.

### Real world studies

Real-world analyses have also highlighted distinct expansion patterns for ide-cel and cilta-cel [14, 43]. Cilta-cel frequently induces more pronounced CD4⁺ CAR T-cell expansion, whereas ide-cel responses are typically driven by CD8⁺ CAR T-cell proliferation and persistence. For ide-cel, greater CD8⁺ expansion correlates with improved clinical outcomes [44]. however, long-term CAR T-cell persistence does not appear to be a prerequisite for prolonged progression-free survival [36].

For cilta-cel, recent studies suggest that expansion of cytotoxic granulysin- or eomesodermin-expressing CAR T-cells as well as activated non-transduced bystander cells is associated with deeper and more durable responses [16]. These same expansion features, however, have also been linked to higher rates of CRS, underscoring the need for continued investigation into how CAR T-cell kinetics contribute to both efficacy and toxicity.

### Recent reports from ongoing trials

At the 2025 Annual Meeting of the American Society of Hematology (ASH), several ongoing clinical trials highlighted continued innovation in BCMA-directed CAR T cell therapy for RRMM. Among these, anitocabtagene autoleucel (anito-cel) demonstrated promising clinical activity, with high overall (97%, 114 of 117 patients) and complete response rates (68%, 79/117), deep MRD negativity (10^-6^: 78%, 53/68), and a favorable acute safety profile, including low rates of severe CRS and ICANS and no reported delayed neurotoxicity or immune effector cell–associated enterocolitis to date [45]. These encouraging efficacy and safety data suggest that the novel D-domain CAR construct may mitigate some known toxicities of prior BCMA CAR T products. However, despite extended clinical follow-up in subsets of patients, detailed data on CAR T cell expansion kinetics, persistence, transcriptional states, and clonal dynamics have not yet been reported, limiting mechanistic insight into the drivers of response durability and long-term safety. Another notable advance presented at ASH 2025 was the dual-targeting BCMA/CD19 CAR T cell AZD0120, which uses a rapid FasTCAR manufacturing platform designed to preserve naïve and central memory T cell phenotypes [46]. In the ongoing DURGA-1 phase 1b/2 study, AZD0120 induced robust in vivo CAR T expansion at both tested dose levels, with peak expansion occurring around day 13 post-infusion. Remarkably, all evaluable patients demonstrated CAR T persistence at day 56, and early persistence was observed even in the 20% (*n* = 5) of the patients previously exposed to BCMA-directed. Despite this robust and sustained expansion, the therapy has so far been associated with a favorable safety profile, with no grade ≥3 CRS, no ICANS, no non-ICANS neurotoxicity, and no late inflammatory complications reported to date. These findings underscore that strong CAR T expansion and persistence do not inevitably translate into severe toxicity, but they also raise critical questions regarding the long-term consequences of sustained CAR T engraftment. Substantial attention was drawn to the emerging concept of in vivo CAR T cell generation, with first-in-human data for KLN-1010 presented as a late-breaking abstract [47]. KLN-1010 employs a modified lentiviral vector engineered to selectively transduce circulating T cells via CD3, enabling in vivo CAR T cell generation without apheresis, ex vivo manufacturing, or lymphodepleting chemotherapy. In the first for treated patients, robust and persistent CAR T cell generation was observed, with CAR-positive cells comprising 22–85% of circulating CD3-positive T cells around day 15 and persisting in blood and bone marrow for 3 months so far in two patients. Notably, one patient developed marked lymphocyte hyperexpansion (absolute lymphocyte counts, ALC > 40000/microliter), which resolved promptly with corticosteroids and without sequelae, highlighting both the potency and the need for careful long-term monitoring of this approach. All treated patients achieved early MRD-negative responses.

These studies illustrate the rapid evolution of BCMA-directed CAR T technologies and their transformative potential. However, long-term follow-up particularly for in vivo CAR T-cell platforms is urgently needed to understand the relationship between pharmacokinetics, response and toxicities with these novel agents.

### The bad – hyperexpansion is associated with off-target infiltration of organs and delayed toxicities

MNTs, particularly parkinsonism, were among the first delayed toxicities described following BCMA-directed CAR T-cell therapy [21]. A recent analysis of the FDA Adverse Event Reporting System (FAERS) provided the most comprehensive overview to date of real-world neurotoxicities associated with ide-cel and cilta-cel [22]. Neurotoxicity linked to cilta-cel included CNP [48]. parkinsonism as well as acute and chronic polyneuropathies. In contrast, ide-cel–associated neurotoxicity more frequently presented with confusion, disorientation, seizures, balance disturbances, and tremors [22].

Cases of GBS, including fatal outcomes, have been reported after cilta-cel [49]. Early manifestations often included acute lower back pain, an early symptom frequently described at the onset of GBS. Besides classical manifestations that include motor weakness, clinical presentations consistent with Miller-Fisher-Syndrome have been described. Accordingly, vigilant monitoring for early signs of GBS is essential, particularly in patients presenting with new or evolving peripheral neuropathies. Prompt involvement of a neurologist is critical to confirm the diagnosis, identify potentially overlapping neuroinflammatory syndromes, and guide appropriate diagnostic testing and therapeutic interventions.

Neurologic toxicity with parkinsonism has been reported in CARTITUDE-1 and 4. Parkinsonism occurred in 1% of patients in CARTITUDE-4 (no Grade 3–4) and in 6% of patients in CARTITUDE-1 (4% Grade 3–4). The median onset of symptoms was 56 days after infusion (14–914 days). Resolution occurred in only 1 of 8 affected patients (13%), with a median time to resolution of 523 days. Median duration of parkinsonism was 243.5 days (range, 62–720 days), including those with ongoing symptoms at death or data cutoff. Notably, the onset of parkinsonism was consistently predated by CRS in all cases and by ICANS in six patients.

Parkinsonism following BCMA-directed CAR T-cell therapy has manifested as movement disorders, cognitive decline, and subtle personality changes often first recognized by spouses or close relatives, who note altered affect or reduced conversational engagement. Patients should be closely monitored for these delayed-onset symptoms, with supportive care and neurological assessment initiated as soon as possible.

To analyze the connection between pharmacokinetics and delayed neurotoxicities, a detailed analysis of CAR T cell expansion in the CARTITUDE 1- 2 and four studies have been reported recently [33]. In total, 355 patients with RRMM received cilta-cel (CARTITUDE-1 (*n* = 97), CARTITUDE-2 cohorts A/B (*n* = 62), CARTITUDE-4 (*n* = 196)). Overall, nine patients (2.5%) developed MNTs and 21 patients (5.9%) experienced CNP. The median time to onset was 22 days (range, 17–101) for CNP and 41 days (range, 19–108) for MNTs.

Because direct measurement of CAR T cells is challenging in real-world settings where specialized assays are typically limited to major tertiary centers ALCs are often used as a surrogate for CAR T-cell expansion [50, 51]. This approach is particularly informative for cilta-cel, as the marked rise in lymphocytes around days 10–14 generally reflects CAR T-cell proliferation rather than expansion of endogenous T cells.

In the analyses from the CARTITUDE trials, patients with MNTs had significantly higher ALC (median 17600 versus 970 cells/µL) and CAR T-cell counts (5520 versus 384 cells/ µL) on day 14 compared to controls [33]. Also, patients with CNP had significantly higher ALC (median 2180 versus 970 cells/µL) and CAR T-cell counts (1230 versus 384 cells/µL) on day 14 compared to controls. Remarkably, patients with MNTs showed a longer persistence of CAR T-cell counts beyond day 100 compared to patients with CNP or controls. CD4 + T-cells were significantly elevated at and around peak CAR T-cell expansion in patients with MNTs or CNP and CD4 + T-cells persisted more often up to day 100 in MNT cases. Real-world observations further indicate that both CRS and ICANS are typically preceded by the peak absolute lymphocyte count (ALCmax) and show a direct correlation with the magnitude of ALC expansion following BCMA-directed CAR T-cell infusion.

In parkinsonism, one proposed mechanism is off-target trafficking of CAR T cells to BCMA-expressing dopaminergic neurons in the caudate nucleus [21]. However, parkinsonism remains rare after BCMA CAR T-cell therapy, and it is almost never observed with BCMA-targeting bispecific antibodies. This raises important questions and suggests that trafficking alone is unlikely to account for all cases. Another possibility is that some patients have increased susceptibility because autoreactive T cells are selected during CAR T-cell expansion. In this model, presentation and recognition of autoantigens could contribute to the development of these delayed neurotoxicities.

Supporting the hypothesis of therapy-triggered autoimmunity, recent reports have described cases of CIDP following cilta-cel treatment [23]. Notably, one patient developed symptoms when the absolute lymphocyte count exceeded 3000/µL. CAR T cells were detectable in both blood and cerebrospinal fluid, yet CD8+ non-CAR T-cells were the dominant population. The authors concluded that CIDP represents a serious complication of cilta-cel and may result from bystander expansion of autoreactive CD8 + T cells rather than direct CAR T-cell–mediated effects.

In addition to delayed neurotoxicity, immune effector cell–associated enterocolitis (IEC-colitis) represents another complication with an incompletely understood pathogenesis and equally challenging clinical management [18, 19, 30, 34]. CAR T-cell infiltration of the GI tract appears to be a consistent feature, but the clonality of these cells has been debated [30]. Our observations, together with those of others, do not uniformly support the notion that IEC-colitis reflects an (oligo)clonal CAR T-cell proliferative disorder. Although some overlap exists with CAR-positive T-cell lymphomas involving the GI tract, most IEC-colitis cases show a predominantly polyclonal CAR T-cell populations.

Off-target trafficking likely contributes to disease initiation. Beyond healthy plasma cells, plasmacytoid dendritic cells have recently been identified as another cell type with relatively high BCMA expression [16]. Both populations are abundant in the GI tract where they support IgA production and antigen presentation and ablation of Ig production can lead to a Common Variable Immune Deficiency colitis-like presentation [52]. Despite this widespread presence, IEC-enterocolitis remains relatively uncommon, again suggesting that expansion or activation of autoreactive T cells may also play a role in disease development.

Another rare but potentially fatal complication linked to CAR T-cell hyperexpansion is HLH or macrophage activation syndrome (MAS), now formally recognized by the American Society for Transplantation and Cellular Therapy [31, 53]. Clinical features include marked hyperferritinemia, hypotension, hypoxia with diffuse alveolar damage, coagulopathy and bleeding, cytopenias, and multi-organ dysfunction, such as renal impairment and respiratory failure. Diagnosing HLH in the CAR T-cell setting remains challenging, as its manifestations overlap substantially with CRS and may be further obscured by baseline abnormalities related to the underlying disease or prior therapies.

Across the CARTITUDE-1 and -4 studies, HLH/MAS occurred in 1% of patients (three of 285) treated with cilta-cel [49]. All cases developed within 99 days of infusion, with a median onset of 10 days (range, 8–99), coinciding with the typical period of maximal CAR T-cell expansion. Each event arose in the context of ongoing or worsening CRS.

### The ugly – clonal expansion of secondary CAR positive T cell lymphoma

The number of patients receiving CAR T-cell therapy for hematologic malignancies, solid tumors, and increasingly for autoimmune diseases continues to rise rapidly [54]. Alongside this growth, reports of secondary malignancies emerging after CAR T-cell treatment have become more frequent [55]. Some of these events, particularly myeloid neoplasms, may reflect prolonged survival in diseases, such as multiple myeloma, where prior exposure to mutagenic therapies has long been linked to an increased risk of secondary primary malignancies [56]. In contrast, certain lymphoid neoplasms can be directly attributed to the infused CAR T-cell product itself. CAR-positive T-cell lymphomas have now been documented following both CD19- [57] and BCMA-directed CAR T-cell therapies [25].

To distinguish physiological CAR T-cell expansion—driven by antigen engagement—from pathological proliferation indicative of a secondary malignancy, we recently outlined four diagnostic criteria [25]. First, the presence of autonomous, dysregulated T-cell proliferation that produces clinical symptoms. Second, molecular evidence of clonal T-cell expansion. Third, an increased mutational burden, typically involving gain-of-function alterations in oncogenes or loss-of-function mutations in tumor-suppressor genes. Fourth, an aberrant immunophenotype of CAR-positive T-cell populations, distinct from that seen in normal post-infusion CAR T-cell kinetics, which can further substantiate the diagnosis of a CAR-positive T-cell lymphoma.

Although several studies have provided deep multi-omic analyses of secondary CAR-positive T-cell lymphomas, their pathogenesis remains incompletely understood. Many reports suggest that selection of a pre-existing clone with hematopoietic mutations, such as *TET2* or *DNMT3A* is more likely than classic insertional mutagenesis. However, a monoallelic integration of a CAR vector into *TP53* has been documented in a single cilta-cel case [26]. The biological diversity and varied clinical presentations of these lymphomas make diagnosis and management difficult. Accurate diagnosis requires detailed phenotypic and genomic characterization to distinguish these cases from other conditions with similar features, such as IEC-colitis. Such analyses may also guide treatment decisions, as shown in a recently described case with targetable CCR4 overexpression [58].

### Management

Pharmacokinetic patterns of CAR T cells in multiple myeloma provide an important diagnostic window that may aid in the detection, treatment, and potentially the prevention of rare but severe toxicities. Our experience, together with published evidence, shows that close monitoring of absolute lymphocyte counts during the first 10–14 days after infusion can serve as a useful surrogate for CAR T-cell expansion [32]. This early signal helps identify patients at increased risk for the toxicities described above [59].

For parkinsonism, other delayed neurotoxicities, and IEC-associated colitis, measuring CAR T cells in peripheral blood and in the involved anatomical compartments is essential. In neurotoxicity, small case series have described the use of conventional chemotherapy [60–62]. either alone or combined with intrathecal treatment [63]. when CAR T cells are detectable in blood or cerebrospinal fluid. However, larger clinical cohorts and prospective trials are still lacking, and a standardized approach to the management of these neurotoxicities has yet to be defined.

Management of IEC-colitis has been guided largely by case reports and small case series, and no standardized approach exists. Infectious causes must be excluded first, as they can mimic CAR T-cell–associated colitis [19]. This step is critical because immunosuppressive treatments that may be appropriate for IEC-colitis could have fatal consequences if an underlying infection is missed. If no alternative explanation is found, biopsies are essential to determine whether CAR T cells are present in the tissue. Endoscopic evaluation should include both the upper and lower GI tract, even when symptoms are limited to the lower GI tract, because the duodenum is frequently involved. An assessment of T-cell receptor clonality is necessary when secondary CAR-positive lymphoma is a concern.

Several therapeutic strategies have shown some benefit in this context. Ruxolitinib [64, 65]. or dasatinib [66–68]. can be used to modulate T-cell activity, while vedolizumab [69]. and infliximab may help control local inflammation [18]. In addition, BCL2 inhibition with venetoclax has demonstrated cytotoxic activity against CAR T cells and may be considered in selected cases [58].

CAR-HLH usually arises after high-grade CRS and often represents a direct transition from severe inflammatory activation. Management beyond tocilizumab and corticosteroids, which are standard of care for high-grade CRS, may include anakinra or cytotoxic agents, such as etoposide or cyclophosphamide [24]. Siltuximab can be considered when CRP and IL-6 levels remain elevated despite repeated dosing with tocilizumab [70]. The interferon-γ–blocking antibody emapalumab, approved for pediatric HLH [71]. has also been used successfully in HLH following CAR T-cell therapy [72]. and is currently under investigation as prophylactic agent in the context of CAR T-cell therapy (e.g. NCT06550141).

A major challenge is that once patients develop fever and markedly elevated inflammatory markers, such as IL-6 and ferritin, the window for effective intervention may already be narrowing. Early recognition, close monitoring, and proactive treatment are therefore essential to prevent rapid clinical deterioration that may become difficult to reverse.

Furthermore, CAR T-cell therapy often induces profound B-cell aplasia, and because prolonged steroid or cytotoxic therapy adds further immunosuppression, patients face a high risk of severe or even fatal infections. Close surveillance for infectious complications is therefore essential. Immunoglobulin replacement should be considered, along with antiviral and Pneumocystis prophylaxis. In selected patients, antifungal and antibacterial prophylaxis may also be warranted[73].

### Open questions and future directions

Many open questions remain regarding the biology of CAR T-cell hyperexpansion as well as persistence and their clinical consequences. Hyperexpansion was initially thought to occur mainly in heavily pretreated patients with substantial tumor burden, but experience in earlier lines of therapy has shown that even patients in deep remission after bridging treatments can develop marked expansion. In fact, some toxicities, such as CNP appear to be more frequent in these earlier-line settings. These observations suggest that antigen load alone does not determine hyperexpansion and that intrinsic T-cell fitness and other patient- or product-related factors also shape this response. Nevertheless, clinical data indicate that achieving a response to bridging therapy is associated with a reduced risk of delayed toxicities [74]. underscoring the importance of reducing tumor burden before CAR T-cell infusion.

It is still unclear why severe off-target effects do not occur more frequently with other anti-BCMA CAR T-cell products or even with bispecific antibodies targeting BCMA. The heterogeneity of published case reports further indicates that these toxicities likely arise from multiple mechanisms rather than a single pathway, which may explain the inconsistent response to current therapeutic interventions. The role of prophylactic approaches in patients with early signs of hyperexpansion is also uncertain. Some groups have suggested using dexamethasone in patients with an absolute lymphocyte count above 3/nL [51, 75]. but prospective evidence is lacking and current clinical experience shows mixed results. More proactive strategies, such as early cyclophosphamide to reduce CAR T-cell abundance or immunomodulatory agents to alter T-cell function, warrant systematic evaluation. Similarly, the optimal management of long-term CAR T-cell persistence remains unresolved. It is not yet known whether eradication of the CAR T-cell population will eventually be necessary to prevent late toxicities in selected patients. Addressing these questions will require carefully designed prospective studies, deeper mechanistic insight, and standardized reporting across clinical centers.
